# Managing borders during public health emergencies of international concern: a proposed typology of cross-border health measures

**DOI:** 10.1186/s12992-021-00709-0

**Published:** 2021-06-21

**Authors:** Kelley Lee, Karen A. Grépin, Catherine Worsnop, Summer Marion, Julianne Piper, Mingqi Song

**Affiliations:** 1 Faculty of Health Sciences, Simon Fraser University, Blusson Hall, 8888 University Drive, Burnaby, BC V5A 1S6 Canada; 2 School of Public Health, University of Hong Kong, UB/F, Patrick Manson Building, 7 Sassoon Road, Pokfulam, Hong Kong; 3 School of Public Policy, University of Maryland, 2101 Van Munching Hall, College Park, MD 20742 USA; 4 Department of Political Science, Northeastern University, 360 Huntington Ave, Boston, MA 02115 USA

**Keywords:** COVID-19, Cross-border health measures, Border management, Travel measures, Trade measures, International Health Regulations, Typology

## Abstract

**Background:**

The near universal adoption of cross-border health measures during the COVID-19 pandemic worldwide has prompted significant debate about their effectiveness and compliance with international law. The number of measures used, and the range of measures applied, have far exceeded previous public health emergencies of international concern. However, efforts to advance research, policy and practice to support their effective use has been hindered by a lack of clear and consistent definition.

**Results:**

Based on a review of existing datasets for cross-border health measures, such as the Oxford Coronavirus Government Response Tracker and World Health Organization Public Health and Social Measures, along with analysis of secondary and grey literature, we propose six categories to define measures more clearly and consistently – policy goal, type of movement (travel and trade), adopted by public or private sector, level of jurisdiction applied, stage of journey, and degree of restrictiveness. These categories are then brought together into a proposed typology that can support research with generalizable findings and comparative analyses across jurisdictions. Addressing the current gaps in evidence about travel measures, including how different jurisdictions apply such measures with varying effects, in turn, enhances the potential for evidence-informed decision-making based on fuller understanding of policy trade-offs and externalities. Finally, through the adoption of standardized terminology and creation of an agreed evidentiary base recognized across jurisdictions, the typology can support efforts to strengthen coordinated global responses to outbreaks and inform future efforts to revise the WHO International Health Regulations (2005).

**Conclusions:**

The widespread use of cross-border health measures during the COVID-19 pandemic has prompted significant reflection on available evidence, previous practice and existing legal frameworks. The typology put forth in this paper aims to provide a starting point for strengthening research, policy and practice.

## Introduction

A cross-border health measure can be broadly defined as action taken to control movement of people (travel) or trade across two or more jurisdictions with the stated intent of achieving a health goal. During the COVID-19 (coronavirus disease) pandemic, the number of countries adopting and impacted by cross-border health measures has been unprecedented. While up to 25% of countries adopted such measures during previous disease outbreaks, virtually all countries have done so during the COVID-19 pandemic [1]. Moreover, countries have adopted a wider range of measures than previously observed and have implemented them in highly varied ways. In turn, studies of cross-border health measures apply diverse, and sometimes inconsistent, terminology to describe these practices. This has reduced the comparability and generalizability of research findings. Media reporting has likewise applied varied and sometimes misleading terms such as ‘border closures’ and ‘travel bans’ despite few jurisdictions actually closing their borders or banning travel. More precisely, a variety of measures have been applied and lifted over time for controlling who travels and under specific conditions. Importantly, this lack of clear and consistent definition persists at a time of substantial debate about the legality and effectiveness of cross-border health measures in response to the COVID-19 pandemic [2–4].

This paper argues that more precise and agreed definition is a starting point for understanding why and how cross-border health measures are used and to what effect. We begin by providing a brief background on the use of these measures during COVID-19, the existing lack of definitional clarity and/or consistency, and the implications for research, policy and practice. To address this gap, we propose six ways to categorize cross-border health measures. We integrate these categories into a proposed typology that can be used, not only to advance research, but to guide decision makers when making choices about the intended purpose, target and implementation of cross-border health measures. We conclude that clear and consistent definition, alongside an agreed typology, is an important starting point for producing generalizable findings, comparative analyses, and evidence-informed responses across jurisdictions. This includes future efforts to revise and improve compliance with the World Health Organization (WHO) International Health Regulations (IHR).

## Background

As the legal framework “to prevent, protect against, control and provide a public health response to the international spread of disease”, the WHO IHR (2005) commits States Parties to act in ways that are commensurate with public health risks and which avoid “unnecessary interference with international traffic and trade” [5]. Cross-border health measures, largely covered under IHR Article 43 (Additional health measures), should only be applied if they meet certain criteria. Such measures are permitted if they are not, among other things, “more restrictive of international traffic and not more invasive or intrusive to persons than reasonably available alternatives that would achieve the appropriate level of health protection,” supported by scientific principles and evidence, and promptly reported to WHO [6]. In the context of COVID-19, however, these criteria have proven somewhat malleable, in part, because of limited, uncertain, and rapidly evolving scientific evidence.

After declaring COVID-19 a Public Health Emergency of International Concern (PHEIC) on 30 January 2020, the IHR Emergency Committee initially recommended against “any travel or trade restriction based on the current information available” [7]. Some States Parties had already adopted travel-related restrictions prior to this declaration. Many more then immediately disregarded WHO’s recommendation, prompting international legal scholars to criticize States Parties for alleged non-compliance with the IHR (2005 [2, 8, 9]. Individuals and groups adversely affected by the restrictions, such as the tourism sector [10] and frontline humanitarian and medical professionals responding to the pandemic [11], called on governments to ease restrictions. As the pandemic worsened, others criticized governments for not applying cross-border health measures earlier and/or more stringently [12, 13], or for easing them prematurely [14]. By March 2020, use of travel-related measures became near universal. Implementation was highly uncoordinated and somewhat chaotic, with cross-border health measures being adopted in highly varying forms, duration, and scope across the world [15–17]. The result has been “a dangerous process of trial and error” [18].

Terminology used by government, media and other commentators to describe this near universal and varied use of cross-border health measures has also lacked clarity and/or consistency. Governments seeking to reassure their domestic populations, that strong action is being taken to reduce the risk of SARS-CoV-2 importation, have often used language suggestive of sealed borders [19–21] or prohibited traffic from high-risk areas [22, 23]. Given their significant social and economic impacts, cross-border health measures have attracted substantial media attention, with terms such as “border closure” [24, 25] and “travel ban” [26, 27] frequently used. In practice, few if any countries sealed their borders or banned travel during the COVID-19 pandemic. These terms are misnomers of actual practice, namely, to restrict selected traffic and manage remaining cross-border movements. They have also obscured the varied practices that countries have followed to achieve this.

Lack of clarity and/or inconsistency in terminology has not helped the increasingly fraught debates about the legality and effectiveness of cross-border health measures. Amid what Kenwick and Simmons describe as growing “border anxiety” during the pandemic [28], the IHR (2005) stipulates that “scientific principles” based on evidence should guide policy decisions. While there is international agreement that decisions about the use of cross-border health measures need to be evidence-informed, systematic reviews conclude that the evidentiary base is limited at best. Most of what was previously known about cross-border health measures is based on studies of pandemic and seasonal influenza, severe acute respiratory syndrome (SARS-CoV-1) and Ebola virus [29]. SARS-CoV-2 combines several features that distinguish it from these previous outbreaks including being a respiratory (versus vector borne) pathogen, having a high reproductive rate (person-to-person transmissibility), and causing many asymptomatic or low-level symptomatic cases [30]. As such, previously held beliefs about cross-border health measures have been questioned in relation to SARS-CoV-2. One systematic review, to assess the effectiveness of “travel-related control measures” during COVID-19 (25 studies), as well as, outbreaks of SARS-CoV-1 and Middle East Respiratory Syndrome (MERS) (11 studies), finds that such measures “may help to limit the spread of disease across national borders” [31]. However, “confidence in these results is limited” given their derivation from assumptions used in modelling studies rather than “real life data”; substantial variation in what measures studies analysed; and the lack of peer review. Our systematic review of domestic and international “travel measures” implemented during the early stages of the COVID-19 pandemic (29 studies) finds that

the adoption of travel measures led to important changes in the dynamics of the early phases of the COVID-19 pandemic. However, most of the identified studies investigated the initial export of cases out of Wuhan, which was found to be highly effective, but few studies investigated the effectiveness of measures implemented in other contexts [32].

We conclude that “[t]here is an urgent need to address important evidence gaps” including the specific cross-border health measures applied, the forms of mobility being controlled, and the context in which they are applied. Most analysis, for example, focus on travel while there has been little study of health-related trade flows during the pandemic. There are also few, if any, comparative analyses of how cross-border health measures have been used in different settings, what factors have influenced their effectiveness at achieving public health goals, and what wider societal effects have resulted.

Efforts to improve the evidentiary base on cross-border health measures have been hindered, in turn, by the lack of clear and/or consistent definition. For example, a decision framework by Zlojutro et al., to optimize border controls for global outbreak mitigation, limits the definition of a border control mechanism to “passenger screening upon arrival at airports (entry screening)” [33]. Habibi et al. exclude screening at ports of entry and exit as “travel restrictions” but include “de facto travel restrictions” notably when “airlines stop flying to places” [2]. Iacus et al. focus their analysis on “air traffic suspension” in “analysing the impact of travel bans on the aviation sector” [34]. Russell et al. define travel restrictions as “any measure that completely or almost completely prevents international arrivals from contributing to local transmission, such as entry bans and compulsory 14-day facility-based quarantines” [35]. Finally, the term non-pharmaceutical interventions (NPI) is widely used in public health research, with different researchers including [36] or excluding cross-border measures [37].

Ultimately, clear and agreed terminology and definition is a critical starting point to advancing shared understandings, cumulative knowledge and ultimately scientific principles on the effective and appropriate use of cross-border health measures during a PHEIC. Outbreaks that involve novel pathogens like SARS-CoV-2, by virtue of being novel, pose challenges for evidence-informed decision making because of knowledge gaps. Nevertheless, it is possible to review and structure best available evidence from previous outbreaks and known pathogens in ways that can inform decision making choices. This begins, once again, with agreed terminology and definition.

## Methodology

The *Pandemics and Borders Project* (https://www.pandemics-borders.org/) is analysing the near universal use of cross-border health measures by countries during the COVID-19 pandemic. For this article, we begin with the WHO Public Health and Social Measures (PHSM) Dataset [38] which collates “data from the main trackers, bringing them into a standard structure and coding them to a common taxonomy.” The trackers currently include the ACAPS government measures dataset [39], Johns Hopkins University Coronavirus Research Centre dataset (a subset of the national and state level dataset) [40], University of Oxford Coronavirus Government Response Tracker [41], and US Centers for Disease Control and Prevention COVID Data Tracker Weekly Review [42]. We then compiled the terminology used in the common taxonomy and glossary of the WHO PHSM dataset on “domestic travel” and “international travel measures” [43]. In addition, we reviewed the terminology used in 29 studies of cross-border health measures applied, during the early stages of the COVID-19 pandemic, identified in our systematic review [32]. Finally, we reviewed detailed government websites on the adoption and updating of cross-border health measures during COVID-19 available on-line from January 2020 to April 2021. These were identified with on-line searches of Google using the keywords “travel”, “trade”, “border*” combined with “ban*”, “restriction*”, “clos*”, “policy” and “measure”. We focused specifically on Canada, Hong Kong and the USA, for which we are conducting detailed case studies on decision making concerning cross-border measures, along with dozens of other countries and subnational jurisdictions listed in the WHO dataset.

Using these sources, we compiled “text segments” or phrases about these measures. We then applied a grounded theory approach to these text segments. As Udo Kelle explains, this approach contrasts with the classical hypothetico-deductive approach “which requires the construction of clear-cut categories and hypotheses before data is collected” [44]. Instead, categories describing specific properties or characteristics of groups of cross-border measures were allowed to emerge from the text segments. While grounded theory calls upon researchers to let categories emerge from data and not to impose preconceived categories, we recognize that our study of previous outbreaks from a largely international relations (politics and economics) disciplinary lens shaped our engagement with the text segments. Potential categories were identified by the lead author and then discussed by all authors for degree to which it described distinct features or characteristics of cross-border health measures. To assess their heuristic usefulness, we checked for empirical evidence and examples of each category by coding a sample of the WHO dataset (entries for a random date). For categories where there is insufficient empirical data to create a clear system of categorization, or where a category could not be parsed into clear subcategories, they were discarded. For example, the potential category of “compliant” versus “non-compliant” (or degree of compliance) with the IHR (2005) was abandoned because of search for categories on features characterizing the measures rather than their relationship to international law. The latter is further complicated by the limited scientific principles against which to determine compliance. Ongoing legal debates about the meaning and practice of IHR compliance, in light of COVID-19, make it impossible to classify specific measures therefore as either compliant or non-compliant. A second discarded category sought to separately characterise measures by the quality or extent to which they were implemented. After considering the diverse ways in which measures have been adopted during COVID-19, we decided instead to add the category restrictiveness characterized by a broad range of subcategories. A total of six categories were confirmed through coding of empirical data as having heuristic usefulness.

To develop the proposed typology, we carefully considered any relationships, required sequencing by time or place, or prioritisation among these six categories. This led to the identification of a hierarchically ordered structure linking the categories together. This typology does not preclude further categories and refinement through further subcategory levels (e.g. types of travel and trade). However, the aim of this initial exercise is to identify major categories that will begin to more clearly define, conceptualize and then advance research, policy and practice on cross-border health measures.

An important caveat to note is that, given the existing lack of clarity and consistency in terminology, the six categories and typology are only intended to be a starting point for increased standardization. This exercise has not been necessary in the past given the more circumscribed use of cross-border health measures, in form and duration, during previous PHEICs [45]. At the time of writing, the COVID-19 pandemic is ongoing and additional measures may yet be applied that are not included in this analysis. Future refinement of these categories and typology may be needed accordingly.

## Results: categorizing cross-border health measures

There have been an unprecedented number and range of cross-border health measures used at different jurisdictional levels during the COVID-19 pandemic. Applying the methodology described above, we identify six categories that offer clearer definition of distinct features of these measures.

### Measure by policy goal

Cross-border health measures have a stated aim of supporting health policy goals such as the prevention and control of disease spread across jurisdictions, reduction of imported health risks, and promotion of access to health-related goods and services. However, it is important to recognize that cross-border health measures can be shaped by a mixture of health and non-health policy goals. Multiple goals are usually at play simultaneously, representing potential trade-offs among different parts of government. For example, the Canadian government announced the cancellation of direct flights to Mexico and selected Caribbean holiday destinations on January 29, 2021 in response to the risk of importing variants of concern. The decision not to ban all international flights, despite the global circulation of variants, reflected a desire to control the adverse economic impacts from reduced travel and trade [46]. Similarly, the easing of travel restrictions by many European countries during summer 2020, to stimulate the tourism sector, increased the spread of SARS-CoV-2 across the region [47]. In some cases, health and non-health goals may come directly into conflict. For example, opposition by some member states of the World Trade Organization (WTO), to a temporary waiver of intellectual property rights over patented COVID-19 vaccines, protects the economic interests of the pharmaceutical industry. In doing so, countries currently with limited access to vaccines are not permitted to manufacture generic versions of vaccines for domestic populations and export to other countries in need [48]. There may also be competing health-related goals at play. A good example is the European Union’s use of export controls to secure vaccine supplies for priority use by member states versus supply to non-EU countries [49]. Finally, governments may claim to apply a cross-border measure for health purposes but be advancing another policy goal. For instance, the indefinite extension of border restrictions by the Trump Administration, on grounds of controlling SARS-CoV-2, was described as an effort “to aggressively use public health laws to reduce immigration” [50].

The co-existence of multiple, sometimes competing, policy goals may help explain choices made about what cross-border health measures have been used during the COVID-19 pandemic, and how they have been applied. In the US, the Trump Administration’s announced “travel ban” on February 2, 2020, applying only to foreign (but not American) nationals who had travelled to China during the previous 14 days, suggests policy choices driven by political rather than public health risk management [12]. When protecting public health is the primary goal, measures may be applied universally and stringently. For example, Australia has strictly limited international arrivals to a quota of nationals, permanent residents and essential travellers, in addition to adopting mandatory testing and enforced quarantine policies with minimal exemptions to prevent virus introduction under a virus eradication (i.e., COVID zero) strategy. By contrast, the UK has adopted targeted restrictions for selected travellers based on an agreed level of acceptable risk of virus importation, a policy intended to balance public health and economic policy goals through a virus suppression strategy (i.e., level of transmission not exceeding healthcare system capacity). Similarly, restrictions on land border crossings between Canada and the US have exempted travel and trade for economic purposes as “essential”. Moreover, the adoption of a measure to achieve a specific public health goal (e.g., prevent importation of infection) might interfere with another public health goal (e.g., cross-border movement of health care workers and carers) in the same jurisdiction. Or a cross-border health measure adopted in one jurisdiction (e.g., restricted export of personal protective equipment, vaccines) can lead to public health harms in another jurisdiction. The potential adoption of so-called “vaccine passports” or “immunity certificates” to enable the lifting of cross-border health measures further illustrates that a measure can lead to or amplify existing social inequities within and across countries.

Moreover, it is also important to note the differential impacts of cross-border health measures on diverse populations. Measures adopted to protect the health of one population group from COVID-19, such as domestic populations, can have detrimental consequences for the health and well-being of mobile populations such as migrant workers, refugees and asylum seekers. During the COVID-19 pandemic, the near universal adoption of travel restrictions by governments has led to millions of migrants being stranded worldwide. Many prevented from returning home must live in conditions which put their health at risk [51, 52]. This points to human rights and ethical concerns regarding the potentially inequitable impacts of cross-border health measures on vulnerable populations.

Overall, the use of cross-border health measures occurs within a complex policy environment. Faced with multiple and sometimes competing goals, decision makers must weigh these against the normative frameworks informing their choices. Scientific evidence should be considered although this may be imperfect and incomplete amid a public health emergency unfolding in real time involving a novel pathogen. The impacts of policy choices on public health and broader economic, social and political goals may thus be unknown and even unknowable.

### Measure by type of movement

A broad range of cross-border measures are applied by different regulatory authorities, to control different forms of movement between one jurisdiction to another for different purposes. These movements generally fall into two broad subcategories: travel-related (i.e. human) and trade-related (e.g. non-human animals, goods and services, information, financial capital). When analysing cross-border health measures, it is useful to distinguish between these two subcategories. The IHR (2005) makes this distinction, defining a “traveller” as limited to “a natural person [human] undertaking an international voyage.” The term “international traffic” encompasses both travel and trade, defined as “the movement of persons, baggage, cargo, containers, conveyances, goods or postal parcels across an international border, including international trade” [53].

The category of type of movement to be controlled will depend on the nature of the risk. If the health risk is a food borne hazard (i.e. biological, chemical or physical), for example, the measure adopted would target relevant trade such as livestock (e.g., bovine spongiform encephalopathy), fresh produce (e.g., salmonella, e-coli) or manufactured product (e.g. lead poisoning) [54, 55]. If the health risk is a communicable pathogen transmitted by direct contact, droplet or airborne, cross-border health measures would target travellers. For vector-borne diseases, travel or trade-related movements may need to be targeted (e.g., spraying, disinfection). These subcategories of travel and trade also help to identify forms of movement that are not currently, but may need to be, controlled. The flow of health-related mis/disinformation via social media across jurisdictions, for example, has come under growing scrutiny given potentially adverse impacts on adherence to public health protocols during the COVID-19 response [56].

Within each of the two subcategories of travel and trade, a further sub-classification can be made between, first, measures that determine whether and how much movement (travel or trade) is permitted, and second, what conditions movement is permitted to occur. The first concerns measures that encompass government policy regarding travel to or from specific jurisdictions which can be an advisory or alert (recommendation) or mandatory restriction. For travel, the latter may take the form of a ban on movement for a selected jurisdiction or population. For trade, the latter may take the form of an embargo on trade with a selected company or country. Measures that determine movement also include actions that impact the logistics of travel or trade notably whether a point of entry is open or closed, or the availability of transport for travel or trade (e.g., cancellation of flights, cruises). The securing of special documentation (e.g., visa) and payment of fees (entry/exit fee, import/export tariff) are further measures that determine movement. Finally, movement can be impacted by limits by volume (e.g., ceilings, quotas) or purpose of movement (e.g., essential versus non-essential). For example, during COVID-19, most countries have permitted the continued movement of what is deemed essential travel (e.g., key workers, armed forces, diplomacy, humanitarian reasons) and essential trade (e.g., medical supplies, food). The import and export of essential supplies such as personal protective equipment, ventilators and vaccines were also the subject to control measures by up to 80 countries [57, 58].

Second, there are measures that determine the conditions under which travel and trade movements are permitted to occur. These measures manage health risks associated with traffic moving across different jurisdictions by identifying the presence of the risk (i.e. screening/inspection, testing, vector surveillance), controlling the onward spread of risk (i.e. contact tracing, quarantine/isolation, vector control, certification), and improving public awareness of risk (i.e. information provision). This distinction provides a potentially first step in determining whether a measure is appropriate or proportionate to the nature of the health risk to be addressed. As stated by the IHR (2005), “measures shall not be more restrictive of international traffic and not more invasive or intrusive to persons than reasonably available alternatives that would achieve the appropriate level of health protection” [6]. For some risks, therefore, such as yellow fever, measures to determine whether movement should occur are unnecessary as vaccination certification is sufficient. During the COVID-19 pandemic, however, multiple measures have been needed simultaneously given the nature of the pathogen and mode of transmission. A summary of travel and trade measures, by whether movement permitted and what conditions movement occurs, is summarized in Table 1.

**Table 1:** Cross-border health measures to control travel- and trade-related movements

	INTERNATIONAL TRAFFIC	
	TRAVEL-RELATED MEASURES	TRADE-RELATED MEASURES
**WHETHER MOVEMENT PERMITTED**	travel advisory or alert (recommendation) or restriction (requirement)	trade advisory or alert (recommendation) or restriction (requirement)
	point of entry by land, air or sea open/closed	point of entry by land, air or sea open/closed
	transport availability	transport availability
	entry or exit restriction	import/export restriction
	visa requirement	licensing requirement
	essential travel documentation	essential goods or services documentation
	ceilings and quotas	import/export quota
**WHAT CONDITIONS MOVEMENT OCCURS**	contact tracing	
	entry/exit fees and surcharges	import/export tariff
	screening	inspection
	testing	testing
	quarantine/isolation	quarantine/isolation
	disease free or vaccination certification	disease free or vaccination certification
	information provision	information provision
	vector surveillance and control [59]	vector surveillance and control

### Measure adopted by public or private sector

Cross-border health measures are, for the most part, officially adopted and applied by public sector authorities operating across different parts (e.g., health, transport, customs and excise, immigration, law enforcement) and levels (international, national, subnational) of government. During the COVID-19 pandemic, as of April 2021, the WHO PHSM dataset suggests most cross-border health measures adopted have been applied by governments. The IHR (2005) sets out commitments by States Parties to use cross-border health measures only under certain conditions as part of overall actions to prevent the international spread of disease. The IHR is then underpinned by a range of national-level governance arrangements, set out in national and subnational public health legislation, which set out the ways that government can and should act.

However, clearer definition of cross-border health measures should include actions taken by private sector entities that influence travel and trade movements (Table 2) but yet are beyond the scope of the IHR (2005). This was apparent during the early stages of the COVID-19 pandemic when many airlines and cruise ship companies acted quickly and even earlier than many governments in restricting travel. For instance, KLM Airlines suspended all flight operations to and from Shanghai and Beijing from 29 January 2020, while the Dutch government introduced border restrictions on 18 March 2020. In early February 2020, the US cruise ship company Royal Caribbean restricted passengers holding Chinese passports regardless of travel history [60]. Many companies reduced, rerouted, or suspended selected services. While initial cross-border measures by private companies appear to have been motivated by efforts to reduce health risks to passengers, many were subsequently based on business decisions as demand declined and it became insufficiently profitable to continue some services. Other measures are motivated by efforts to reduce the health risks of travelling during a pandemic, as a means of encouraging consumer uptake and resuming business. Thus, the private sector has been at the forefront of developing pre-departure and on-board protocols including hygiene practices, wearing of face coverings, and testing. The private sector has also conducted or sponsored research to assess risks associated with travel [61]. Some airlines have created incentives to travellers such as price reductions and free travel insurance. For the most part, these actions are governed by individual company policies and lay outside the authority of the IHR (2005). Table 2 provides examples of cross-border health measures used by the private sector during COVID-19.

**Table 2:** Cross-border health measures applied by private sector

MEASURE	EXAMPLES
restricted access to mode of transport	flight cancellations, cruise ship cancellations
health screening	passenger screening
testing	testing contractor
certification	vaccine passport scheme
financial incentives/disincentives	reduced pricing, free health insurance

Alongside recognition of measures taken by both the public and private sector, analysis of the effective use of cross-border health measures should take account of the interconnectedness of these actions. For example, the US government’s restrictions on travel to and from Wuhan, China is believed prompted by airline flight cancellation [12]. Conversely, the adoption of pre-border testing requirements by many governments led some airlines and airports to provide convenient and even complimentary testing facilities for travellers. Private contractors have been hired to implement certain measures mandated by government (e.g., testing, quarantine enforcement). Carriers have been punished by some governments for failing to comply with such measures. In Hong Kong, for example, if an airline violates travel restrictions (e.g., allowing someone to board without the appropriate negative test certificate), or if too many travellers subsequently test positive, the airline is banned from inbound travel for 14 days. As a result, KLM, Emirates, and British Airways were all banned at one point, and Air India and Nepal airlines were repeat offenders [62]. Where governments have failed to act swiftly enough, such as providing testing and vaccination of employees in the US, the private sector has stepped into the breach [63].

Alternatively, a disconnect between public and private measures has been evident in some countries, whereby a government strongly advises against but does not prohibit non-essential travel, while businesses such as airlines and hotels continue to advertise holidays, thus confusing the public about official restrictions. Travel by numerous public officials as private citizens for holidays and other non-essential reasons despite these advisories have further added to public confusion and distrust [64]. In Canada, the federal government and airlines sought to better align messaging and practice with an agreement announced in January 2021 to cancel direct flights to and from selected holiday destinations in Mexico and the Caribbean [65]. However, indirect flights continued to operate for these destinations by other airlines. Overall, defining cross-border health measures requires an understanding of the roles of both the public and private sector, and how the relationships between them influences cross-border governance.

### Measure by level of jurisdiction applied

Analyses of cross-border health measures prior to COVID-19 focused on travel and trade between two or more countries (i.e., international borders) [31]. However, insufficient account has been taken of their use at different levels of jurisdiction. Studies of travel-related restrictions, in particular, focus on national-level measures or have conflated different levels [32]. As a result, it is difficult to separate out the effectiveness of measures applied at a particular level when measures are simultaneously applied at more than one level. Lack of distinction by jurisdiction also undermines the accuracy of comparative analyses.

As the world has become more interconnected from the late twentieth century, traffic volumes have significantly increased. Thus, health measures sit alongside varied national regulation of travel and trade between sovereign states. International agreements set out how States Parties apply these measures in a standardized and coordinated way. For example, the WTO administers international treaties setting out agreed conditions to facilitate the movement of trade and investment. The IHR (2005) sets out commitments by States Parties to coordinate use of cross-border health measures to prevent and control the international spread of disease. The Codex Alimentarius is a collection of standards, guidelines and codes of practice established by the Food and Agriculture Organization and WHO to protect consumer health and promote fair practices in food trade [66].

However, clear definition of cross-border health measures should also take account of their use at other levels of jurisdiction both above and below the state (Table 3). There are bilateral and regional arrangements, encompassing selected countries, relevant to health-related cross-border movements (e.g., European Union, Schengen Area). During non-pandemic times, regional agreements regulate the cross-border movement of patients [70], health care workers [71], and financing. During the COVID-19 pandemic, bilateral agreements have been reached to govern travel across land borders between countries (e.g., US-Canada, US-Mexico) [72]. Regional arrangements to establish “air bridges” and “travel corridors” have proliferated [73]. While most involve two or more sovereign states, some combine a subnational area with sovereign states (e.g. Trans-Tasman Travel Bubble). Amid concerns about limited vaccine supplies, Germany called on potential restriction of exports from the European Union [74].

**Table 3:** Examples of cross-border health measures by level of jurisdiction for COVID-19

	TRAVEL	TRADE
**INTERNATIONAL (ALL COUNTRIES)**	Quarantine measures for travellers under the IHR (WHO)	Codex Alimentarius Commission Standards (WHO/FAO)
**REGIONAL/BILATERAL**	European Council Recommendation on coordinated approach to restriction of free movement [67] US-Mexico agreement to limit non-essential travel UK travel corridors to 50 countries Tasman Bubble (Australia/New Zealand)	Restrictions by European Union on vaccine exports
**SUBNATIONAL (STATE/PROVINCE)**	Atlantic Bubble (Canada) state-level travel restrictions (Australia)	US federal control of PPE supplies to individual US states
**MUNICIPAL**	Wuhan lockdown Big White Ski Resort, BC limits bookings to residents (Canada) [68] Gombe commune, Kinshasa lockdown (DRC) [69]	
**MARKET**	Airline requirements for passengers on selected routes (e.g., testing) Cancellation of cruises in selected regions	Purchase agreements for COVID-19 vaccines

During COVID-19, domestic travel restrictions are increasingly recognized as an important means of controlling spread of the coronavirus [75]. Cross-border health measures have been extensively used to control movements across internal or domestic (subnational jurisdictions) borders. In the UK, travel advisories sought to limit non-essential travel between the four nations (England, Scotland, Wales and Northern Ireland). Tiering of areas in England (defined by a mix of cities, counties and subregions), based on “five epidemiological indicators” (e.g., coronavirus infection case detection rates, positivity rate), sought to limit travel between higher and lower risk areas between November 2020 and January 2021 [76]. Travel restrictions have been used extensively by Australian states and territories to control domestic spread of the coronavirus [77]. In Canada, the so-called “Atlantic bubble” formed from June–November 2020 allowed unrestricted travel by residents among four Canadian provinces, and restricted travel by non-residents from other provinces [78, 79]. In the US, travel restrictions have localized by state, municipality and even neighbourhoods (e.g., New York City) [80]. There have been fewer efforts to control subnational trade. One example is the US where, amid surging cases of COVID-19, it was reported that the Trump Administration blocked PPE supplies to several states including Michigan and Massachusetts [81]. Shortages in essential supplies (e.g., PPE, ventilators) in the US during the early stages of the pandemic led to reports of diversion of placed orders of selected states by the federal government [82].

Finally, when analysing cross-border health measures, further complexity can arise when arrangements combine multiple jurisdictional levels. For example, during the early stages of the pandemic, different countries adopted a mixture of restrictions on travellers from the city of Wuhan, Hubei Province and China as a whole. Alternatively, travel restrictions may exempt daily commuters between a country and a subregion of a neighbouring country (e.g., Switzerland and bordering regions in France; day workers crossing the bridge from Johor, Malaysia into Singapore). Travel restrictions seeking to control the spread of SARS-CoV-2 and variants of concern have simultaneously targeted selected cities, countries, and regions [41]. For example, Japan used a targeted subnational approach vis-a-vis parts of northern Italy in March 2020 [83] which mirrored earlier restrictions for travellers from parts of China, Iran, and South Korea [84]. Concerns about the spread of the P1 variant led the UK government to ban flights from South America and Portugal [85]. Jurisdictions with special or disputed legal status also require specific arrangements. Movements between Hong Kong and mainland China, for example, are treated as somewhere between international and domestic given the former’s status as a special administrative region, with its own immigration policies, while still part of the country. A similar situation exists regarding stricter travel restrictions adopted by New Caledonia, a French Overseas Territory, and the rest of France, which only permits French citizens arriving on a select route (via Japan) and then only after undergoing quarantine. There are also now thousands of special economic zones worldwide to facilitate trade and investment, located within a country’s national borders, but governed separately. Most complex, perhaps, are measures taken by private sector actors according to market-based jurisdictions combining mode of transport (e.g., cruise itinerary) and geographical location (e.g., Asia). For jurisdictions such as these, measures adopted during COVID-19 may reflect the specific political, economic or legal status of these jurisdictions.

### Measure by stage of journey

Cross-border health measures can be categorized by the point of application along three main stages of a journey – pre-border, at-the-border and within-the-border. The stage at which a measure is applied can depend on considerations such as administrative convenience, cost, logistics and nature of health risk. In general, measures determining whether movement is permitted are applied at the pre-border stage to minimise denial of entry at a border. Thus, travel and trade are impacted by recommendations or requirements to avoid certain jurisdictions, the opening or closure of points of entry, and availability of transport. Where specific procedures must be followed or documentation needs to be obtained, which can be time-consuming and costly, it is also more appropriate to administer these at the pre-border stage. This includes assembling evidence of eligibility to travel or trade, screening/inspection, health certifications, testing and pre-travel quarantine. In December 2020, for example, the Chinese government introduced the requirement that foreign nationals undergo nucleic acid and IgM anti-body tests and complete a Health Declaration Certificate two days before boarding [86]. For the safe importation of live animals into the European Union, to avoid the transmission of diseases, health certificates must be obtained beforehand, signed by an official veterinarian of the competent authority of the exporting non-EU country guaranteeing that the conditions for import into the EU have been met [87].

At-the-border measures are applied when a traveller or trade physically reaches a point of entry. These include checking necessary documentation (e.g. visa, proof of essential travel, vaccination or negative test certificates), further testing or vaccination (e.g. yellow fever), screening/inspection and quarantine/isolation; and payment of additional fees, fines or penalties. Vector control through disinfection and disinsection procedures may be applied. Quarantine of non-humans (i.e. pets, wildlife, livestock) is applied at a border by customs officials who check documentation, inspect the animals, and keep them in quarantine for a requisite period depending on the type of animal until their release from customs [88].

Finally, some measures are applied within-the-border to extend the management of health risks after entry into a jurisdiction. Quarantine of humans may be applied at-the-border but, strictly speaking, is administered after a traveller enters a jurisdiction. During the Ebola virus outbreak in 2014, for example, returning travellers to the US were quarantined at some airports, although this technically occurred on American soil [89]. During the COVID-19 pandemic, mandatory quarantine of international and some domestic arrivals after entry has been carried out at designated sites or places of resident. Further screening/inspection and testing of travellers and trade after entry may occur. In England (but not the rest of the UK), international travellers must test prior to departure (within 72 h), and days two and eight after arrival [90]. Other countries require further testing at varying number of days after arrival. A summary of travel and trade measures by stage of journey is provided in Table 4.

**Table 4:** Cross-border health measures by stage of journey

	PRE-BORDER	AT-THE-BORDER	WITHIN-THE-BORDER
**TRAVEL**	travel advisory or alert (recommendation) or restriction (requirement)		
	point of entry by land, air or sea open/closed		
	transport availability		
	entry or exit restriction		entry or exit restriction
	ceilings and quotas		
	visa requirement	visa requirement	
	essential travel documentation	essential travel documentation	
	disease free or vaccination certification	disease free or vaccination certification	
	travel insurance coverage for health care		travel insurance coverage for health care
	screening	screening	screening
	testing	testing	testing
	quarantine/isolation	quarantine/isolation	quarantine/isolation
	entry/exit fees and surcharges	entry/exit fees and surcharges	
		fines and penalties	fines and penalties
			contact tracing
**TRADE**	trade advisory or alert (recommendation) or restriction (requirement)	customs inspection	health inspection
	point of entry by land, air or sea open/closed		
	transport availability		
	licensing requirement	seizure, detainment and/or destruction	seizure, detainment and/or destruction
	technical requirements (e.g. labelling)	fines and penalties	fines and penalties
	levy and payment of tariffs	levy and payment of tariffs	levy and payment of tariffs
	vector surveillance and control	vector surveillance and control	vector surveillance and control
	quota (maximum volume of travel or trade)		
	inspection	inspection	inspection
	testing		

The categorising of cross-border health measures using a full journey perspective encourages a more comprehensive and integrated approach to managing health risks associated with cross-border mobility. In some cases, measures may be required at multiple stages of a journey (e.g. screening, testing and quarantine). Testing at multiple points to reduce any residual risk, for instance, is needed because of ongoing risks of exposure, the course of infection (i.e. insufficient viral load for detection, incubation period) and false negative results. This approach is illustrated by Australian travel policy during the COVID-19 pandemic which covers measures from pre-travel to the end of the journey. Travellers to Australia must first be deemed eligible, specify whether they will arrive via a red or green zone (i.e. level of risk), self-screen for symptoms, take a PCR test and test negative within 72 h of departure, and pre-book an available slot (within the quota limit) at a designated quarantine hotel (if required) prior to departure. Upon arrival, the traveller must provide proof of a negative test result, be screened, tested and, if from a red zone, sent to a government designated quarantine facility for a minimum of 14 days. During quarantine, further tests will be administered at 10–12 days. If negative, the traveller will be permitted to enter Australia but must abide by all public health rules including any inter-state travel restrictions. If tested positive at any time, they must remain in isolation [91].

This full journey approach also supports efforts to manage the costly and challenging logistics of implementing cross-border health measures for high volumes of travel and trade. Given finite resources, decision makers may consider how measures applied at one stage, such as vaccination and testing, can potentially reduce the need for the same measures at another stage. For instance, if a traveller provides proof of a yellow fever vaccine received at the pre-border stage, there is no need for vaccination, testing or quarantine at the point of entry. The increased use of pre-border measures can reduce the administrative burden at the point of entry, and reduce the need to implement substantial capacity at-the-border and potential bottlenecks that hinder cross-border flows. These benefits, however, need to be weighed against potential increased risks of reduced measures such as lower detection of travellers that become infectious at a later stage of their journey. During COVID-19, many governments require travellers to use on-line apps to register required documentation and pre-book reservations in quarantine facilities. If a government is seeking to discourage non-essential travel, pre-border measures can act as a disincentive by shifting the administrative and financial burden onto these travellers. Finally, effective use of pre-border measures can play a valuable preventative role, by screening out or reducing imported health risk. For example, regular inspections at the point of manufacture or processing can enhance the safety before imported products or produce arrive at their destination. Any assessment of the effectiveness of cross-border health measures applied during COVID-19 needs to take account of how they have been used for each of these three stages of a journey.

### Measure by degree of restrictiveness

While the terms “travel ban” and “border closure” have come to be commonly used during the COVID-19 pandemic, in practice, cross-border health measures vary widely in degree of restrictiveness. The least restrictive are advisories or warnings by a government seeking to raise awareness of a potential health risk from travel or trade. Certain precautions may also be recommended to manage the risk. For travel, this might be delaying or avoiding travel, vaccination or bringing essential supplies. For trade, this might involve not purchasing or consuming a product (e.g. contaminated food product), issuing a recall or disposal. The most restrictiveness measures are prohibitions on certain travel or trade.

The two most common subcategories of traveller status used are citizenship or residency, and purpose of travel (e.g. essential versus non-essential). Travel restrictions may also be targeted at travellers from selected source countries deemed to be a particular risk to health. Importantly, governments may also adopt measures that exempt or ease travel restrictions based on certain categories of traveller [92] or source country. Travel on humanitarian grounds (e.g., refugee, direct family member) or economic benefit (e.g., students, essential worker), or travel from selected jurisdictions deemed safe from health risks, may be given exemptions and even “fast tracked”. Trade-related restrictions can also range from advisories, such as consumer alerts on potentially harmful products, to bans on certain categories of goods or from a target source country. During the COVID-19 pandemic, many countries eased certain trade policies to facilitate the importation of necessary health supplies and equipment, while others tightened export controls to keep needed health and medical supplies and equipment for national use.

**Fig. 1:**
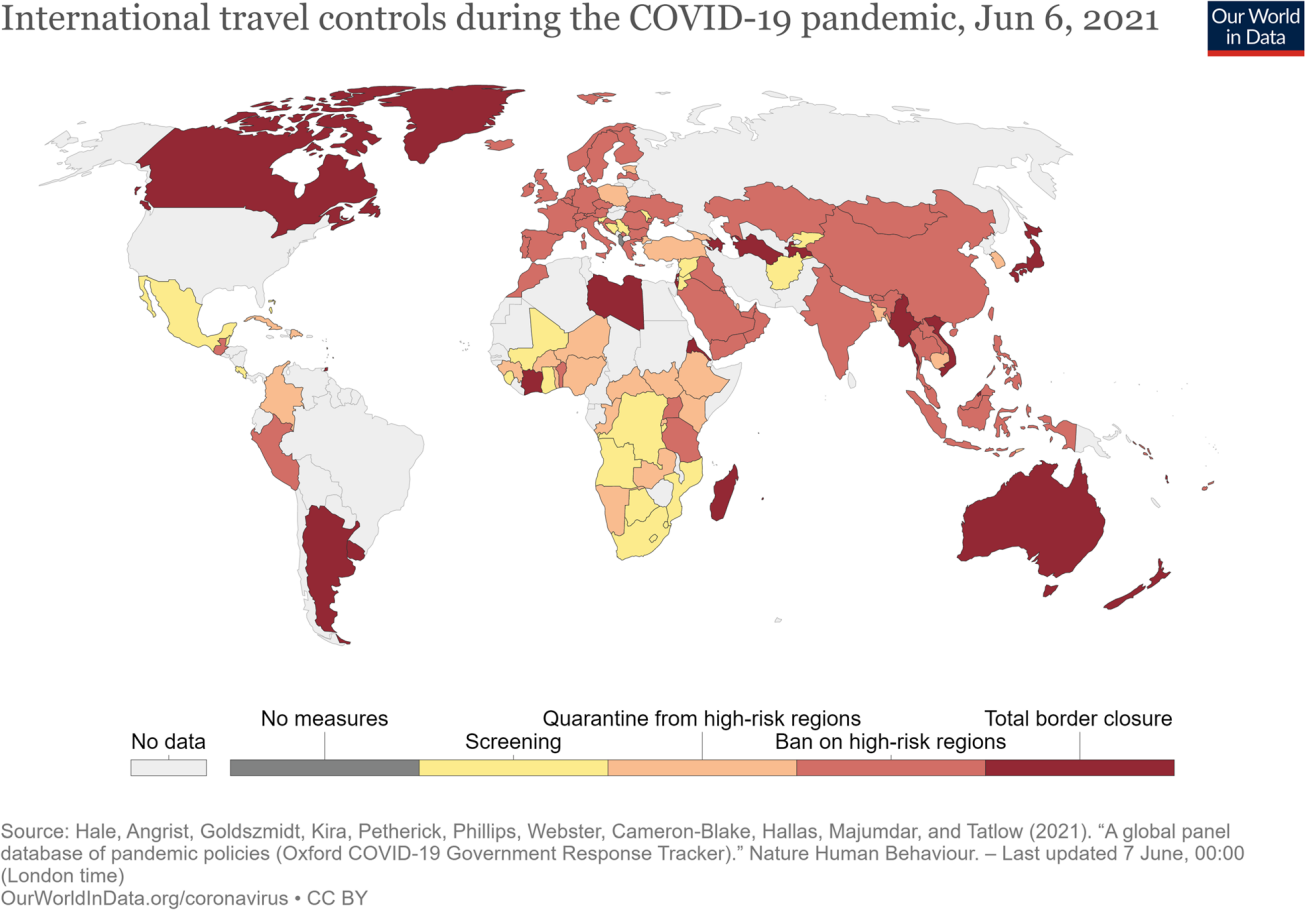
International travel controls during the COVID-19 pandemic, Dec 10, 2020. Source: https://ourworldindata.org/policy-responses-covid

**Fig. 2:**
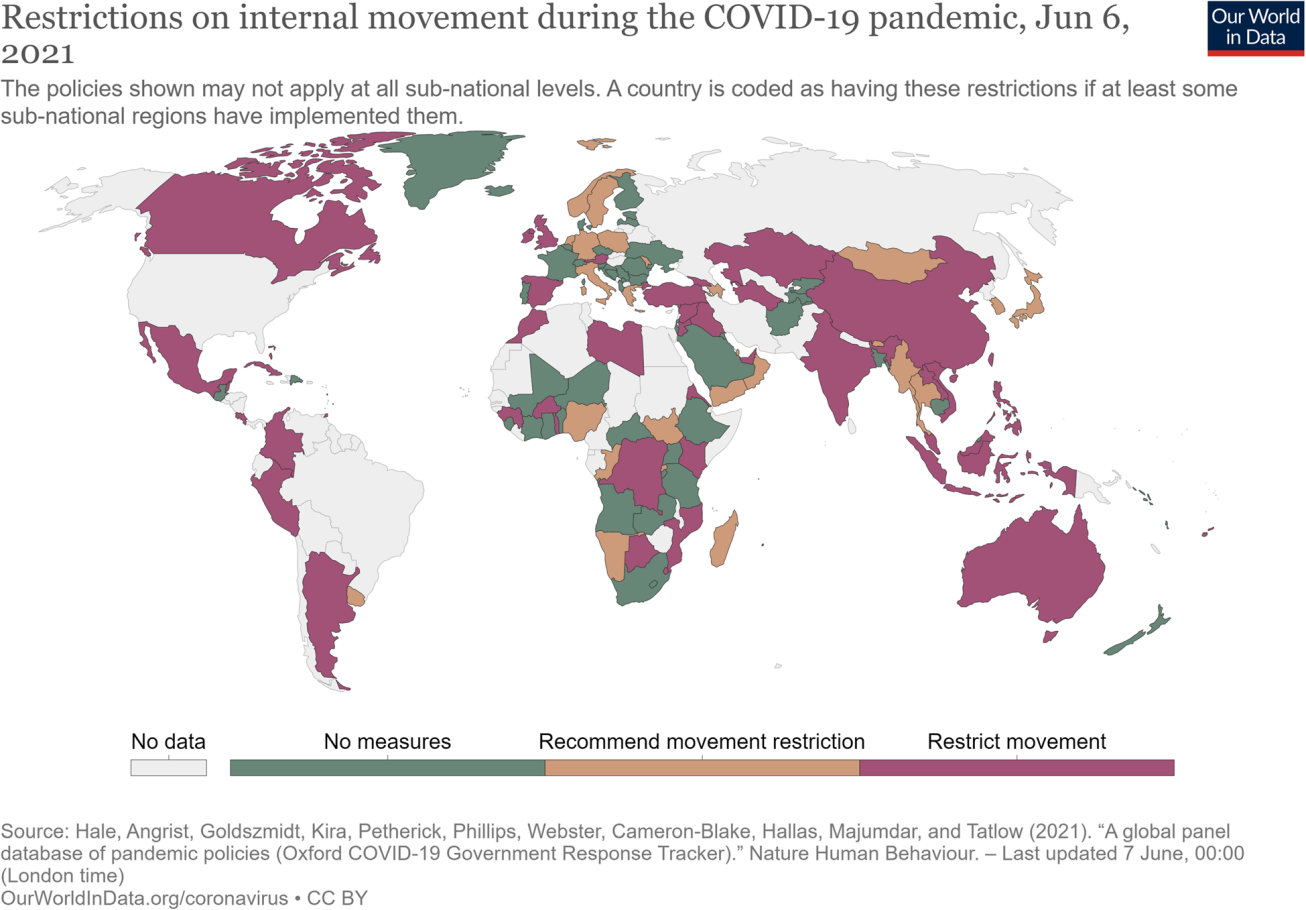
Restrictions on internal movement during the COVID-19 pandemic, Dec 10, 2020. Source: https://ourworldindata.org/policy-responses-covid

Given the above, efforts have been made to categorize selected cross-border health measures by their degree of restriction during the COVID-19 pandemic. The Oxford Coronavirus Government Response Tracker, for example, collects data on 19 indicators, and records their stringency at given points in time [41]. For international travel controls, stringency ranges from no measures to total border closure (Fig. 1). For internal (domestic) movements, stringency of measures are categorized as no measures, recommended movement restriction and restrict movement (Fig. 2). However, only a limited number of the travel-related measures listed in Tables 1 and 2 are included in this analysis. Similarly, the International Air Transport Association (IATA) COVID-19 Travel Regulations Map categorizes countries as Totally Restrictive, Partially Restrictive or Not Restrictive, with no information provided on what measures are assessed or how [93].

Building on these efforts, we argue that any comparative assessment of the use of cross-border health measures needs clear criteria to assess restrictiveness or stringency by type of movement and measure over time and place. On type of movement, the extent to which a measure impedes the movement of the targeted travel or trade is the overall criteria. In addition, specific measures vary in their restrictiveness, as well as, how specific measures are implemented can vary in restrictiveness (Table 5). For example, screening (e.g. health declaration, temperature check) would be considered less restrictive to travel than quarantine. In addition, specific ways of implementing quarantine (i.e. duration, location, voluntary versus mandatory, liability for cost) influences stringency. Restrictiveness is also cumulative, with the use of multiple cross-border health measures simultaneously capable of creating greater stringency. In all cases, degree of exemptions from the measure directly impacts on restrictiveness. In Canada, there are many exemptions from restrictions on international arrivals by non-nationals and non-permanent residents including essential workers, direct family members, holders of study visas, military personnel, US citizens travelling to Alaska, and additional individuals exempted by the government [106]. Many of these categories are also exempt from the 14-day quarantine requirements. In Hong Kong, there are few exemptions to entry and quarantine permitted such as business executives of companies listed on the Hong Kong Stock Exchange travelling to and from the Chinese mainland [107].

**Table 5:** Examples of restrictiveness of selected cross-border health measures during COVID-19 pandemic

MEASURE	FACTORS AFFECTING RESTRICTIVENESS	EXAMPLES
screening	target population, stage(s) of journey, screening method, frequency, intended data use, exemptions	Low Germany: Carriers arriving from China, South Korea, Japan, Italy and Iran required to report health status of passengers before entering Germany. Information on Disease prevention distributed to passengers (28 February 2020) [94] Medium Taiwan: All arrivals required to complete health declaration and provide travel and contact history if visited China, Hong Kong or Macao within 14 days before entry (12 February 2020) [95] High Benin: All arrivals coming from countries affected by COVID-19 (including countries with only a single case of COVID-19) must identify themselves using hotline. Such persons must self-isolate for 14 days and may be subject to additional screening and/or relocation to a quarantine facility (10 March 2020) [96].
testing	target population, stage(s) of journey, timing, frequency, type of test, liability for cost, exemptions	Low United Kingdom: No testing requirements for international arrivals until introduced on 18 January 2021 [97]. Medium Norway: International arrivals must present proof of negative test for coronavirus taken less than 24 h prior to entry. Five categories of population are exempted (30 January 2021) [98] High Germany: Two-test strategy which provides for mandatory testing in connection with entry and, voluntary testing for early termination of quarantine at the earliest from the fifth day after entry. People who enter Germany after staying in “high incidence areas” and “virus variant areas” in the last 10 days before entry is obliged to bring proof that they are not infected with the SARS-CoV-2 coronavirus upon arrival. The test must be carried out at least 48 h before entry. If the persons could not obtain evidence, carriers can carry out or have a test carried out before departure. The smear for this test by the carrier may be made no more than 12 h before departure (2 February 2021) [99]
quarantine	target populations, stage(s) of journey, length, location, voluntary versus mandatory, liability for cost, method of enforcement, exemptions	Low USA: CDC recommends international arrivals self-quarantine for 7 days after travel (18 February 2020) [100] Medium Canada: International arrivals by air required to undertake up to 3-day mandatory quarantine in designated hotels at traveller expense. If PCR test upon arrival negative, remaining quarantine completed at home with limited monitoring. International arrivals by land and sea required to undertake 14-day quarantine with limited monitoring (22 February 2021) [51] High Australia: Mandatory enforced 14-day quarantine introduced for all international arrivals (citizens primarily) who must pre-book one of limited slots in designated facility. Travellers pay cost of quarantine. Limited categories of exemption permitted to quarantine at home (29 March 2020) [101]
evidence of eligibility to travel	target populations, categories of eligibility, cost of entry visa	Low USA: Foreign nationals who travelled to China within past 14 days banned from entry (2 February 2020) [102] Medium Estonia: Foreign workers in agricultural sector allowed to extend short-term work permit. Workers from other sectors excluded (21 April 2020) [103] High Philippines: Non nationals denied entry with exception of crew members, government and international organization officials, and “uniformed personnel for official business” (20 March 2020) [104] Australia: International arrivals limited to Australian nationals and foreign nationals travelling for essential reasons. Cap of 1475 arrivals per day introduced (4 July 2020) [105]

Importantly, degree of restrictiveness should not be conflated with effectiveness in achieving a stated health goal. Indeed, increased restrictiveness is not the same as greater effectiveness. The appropriate degree of stringency will depend on the health risk posed, including the nature of the pathogen, and specific context for implementation of the cross-border health measure. For screening, in particular, a systematic review of evidence on COVID-19 studies to May 2020 found “[o]ne-time screening in apparently healthy people is likely to miss people who are infected” [108]. For example, temperature screening may be stringently enforced for all travellers, but evidence suggests it offers limited public health benefit during previous pandemics [109]. Analyses of the relationship between degree of restrictiveness and effectiveness will need to account of a complex range of factors such as policy change over time, evaluations of policy implementation, outbreak dynamics and health outcomes.

## Discussion: a proposed typology of cross-border health measures

**Fig. 3:**
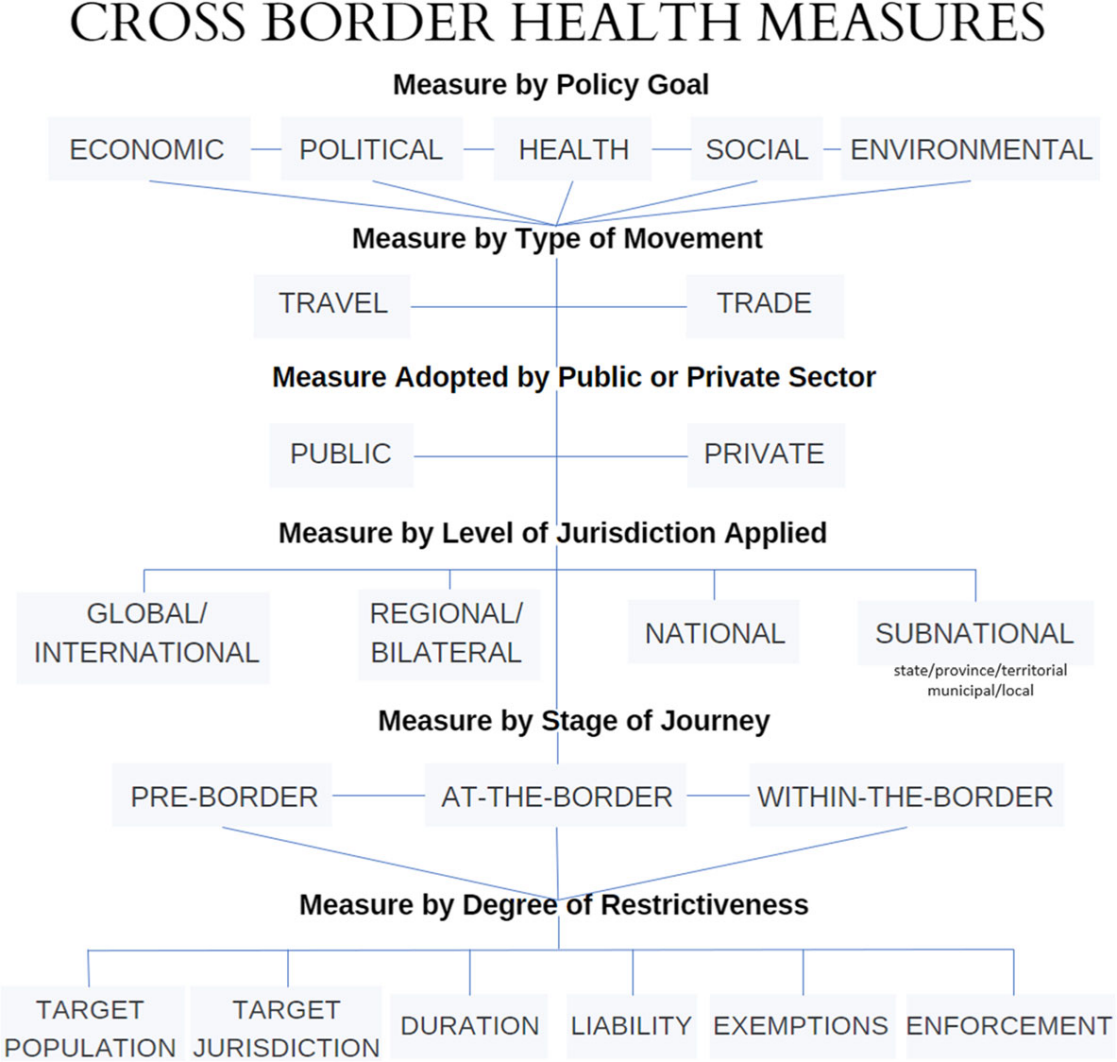
Proposed typology of cross-border health measures

As far as we are aware, this is the most comprehensive effort to develop categories for defining distinct features of cross-border health measures. Bringing together the six categories, Fig. 3 provides a typology of cross-border health measures by type of movement, policy goal, level of jurisdiction, stage of journey, public versus private sector use, and degree of restrictiveness. The typology sets out the many choices to be made when considering border management. This begins with identifying the policy goal(s) to be achieved, recognising that health and non-health policy goals co-exist and influence the choice of measures, how they are implemented, and whether they are ultimately effective. This acknowledges that there will generally be trade-offs, limitations, and compromises to navigate whenever cross-border health measures are considered.

This typology is useful in several respects. First, current research is characterized by varying and conflicting terminology and often imprecise definition, thus reducing the generalizability of findings and comparative analyses across jurisdictions. Efforts to determine the effectiveness of cross-border health measures, in controlling the spread of disease, are challenged by differences in practice and ways of describing them. This typology offers a framework for identifying the specific measures used in a standardized way. It encourages researchers to describe measures with greater specificity and in ways that enable comparability across the six categories. The need for comparative analyses is especially important given growing evidence that “[i]nternational travel was directly associated with the mortality slope and thus potentially the spread of COVID-19. Very early restrictions on international travel should be considered to control COVID-19 outbreaks and prevent related deaths.” [67]

Second, and relatedly, by supporting clear and consistent definition of cross-border health measures, the typology can facilitate research into the varying effects *and* externalities of such measures. Rather than assuming the possible benefits and harms of cross-border health measures, our approach encourages explicit analysis of not only public health effects, but also social, economic, ethical, and political externalities. For example, the social and economic toll of cross-border health measures may disproportionately harm vulnerable groups, countries, and communities, provide a convenient excuse for governments to take discriminatory trade and immigration measures, or create a false sense of security that may detract from other response measures [110–113]. These externalities require explicit investigation that is not possible without clear and consistent definitions of cross-border health measures. Public health benefits may still predominate, but these should not be seen in isolation. Enhanced understanding of externalities can inform harm reduction policies and more accurate assessments of the political implications of a range of cross-border health measures for governments.

Third, the typology aims to support decision-making on the use of cross-border health measures. During the COVID-19 pandemic, policy debates have often focused on whether or travel restrictions work to prevent coronavirus spread. Instead, this typology supports understanding of the complexity of choices faced when thinking about the purpose of such measures, the broad range of measures available, the ways that might be applied singly or in combination, and the importance of timing and context. This shifts decisions, from a binary question of whether or not to use cross-border health measures, to what type of movement may need to be controlled and what conditions need to be introduced to manage an identified health risk. This typology also locates cross-border health measures within the broader context of border management for different, and sometimes competing, policy goals. It is this complexity which explains what Petersen and colleagues call a “dissonance between scientific advice and political realities” [114]. Public communication, when implementing such measures, may also be clarified in ways that enhance compliance.

Fourth, by aiming to strengthen research and policy, the typology may encourage more coordinated use of cross-border measures across jurisdictions. Evidence from previous PHEICs shows that effective global responses are facilitated by coordinated action across countries. However, “there is no recognized coordinating body to disseminate timely, consistent, reliable and authoritative information and best practices to all stakeholders” [115]. During the COVID-19 pandemic, there has been limited coordination, with countries using their own mixture of measures applied in different ways. The costs of uncoordinated action are likely to include increased disease transmission risks, prolonged outbreaks and unnecessary economic and social impacts [116, 117].

Finally, the typology can inform future efforts to revise the IHR (2005). The IHR (2005) is the legal framework for the use of “additional health measures” by States Parties based on “scientific principles.” The typology would support more precise definition of additional health measures, research to identify best practices, and thus the development of scientific principles to guide action. Moreover, the typology may inform future efforts to revise the IHR (2005) by revealing cross-border health measures not currently covered under their remit. Many forms of trade are not currently subject to the regulatory authority of IHR despite having implications for the international spread of disease. For example, export controls of PPE and vaccines during the COVID-19 pandemic fall beyond the authority of the IHR (2005).

The typology also supports an alternative approach to assessing compliance with the IHR, beyond solely legal interpretations of substantive adherence to Article 43 provisions, such as “shall not be more restrictive of international traffic and not more invasive or intrusive to persons than reasonably available alternatives that would achieve the appropriate level of health protection,” and “shall base their determinations upon…scientific principles [and] available scientific evidence” [6]. Clearer definition of what constitutes “additional health measures”, empirical analyses of their public health and broader impacts, and fuller understanding of the complexity of empirically and normatively-based choices involved when applying and lifting such measures, suggests scientific principles for all types of outbreak events (notably for emerging pathogens) and social settings over time may remain elusive. Instead, compliance could be additional assessed by the application of a decision instrument, informed by best practice and the transparent consideration of agreed normative values, applied over time during an unfolding event. Governments might thus demonstrate compliance through procedural due process, applying such a decision instrument, to guide real-time choices. Use or non-use of cross-border health measures could thus be deemed compliant with the IHR through some combination of the letter and spirit of the law [118]. The capacity to assess both will still require significant improvements in the evidentiary base regarding the effective use of such measures. A critical starting point for this important research, which this paper seeks to advance, is clear and consistent definition and conceptualization of cross-border health measures.

## Conclusion

The widespread use of cross-border health measures during the COVID-19 pandemic has prompted significant reflection on available evidence, previous practice and existing legal frameworks. All are found wanting, particularly given insufficiently clear and/or consistent definition about the subject at hand. The typology put forth in this paper aims to provide a starting point for strengthening research, policy and practice. By encouraging a fuller understanding of border management using six categories of measures, the use of cross-border health measures during future PHEICs can be more evidence-informed, capable of navigating policy complexity including managing trade-offs and externalities, and ultimately appropriate and effective in protecting and promoting population health. We argue that these are critical foundations to a more coordinated approach to the use of cross-border health measures across jurisdictions.

## Data Availability

Not applicable.
